# Structural and Mechanical Characterization of Collagen-Hyaluronan Hydrogels Used to Study Cancer Cell Invasion through the Bladder Wall

**DOI:** 10.1021/acsbiomaterials.5c00136

**Published:** 2025-05-15

**Authors:** Sara Metwally, Justyna Śmiałek-Bartyzel, Joanna Pabijan, Małgorzata Lekka

**Affiliations:** Institute of Nuclear Physics, Polish Academy of Sciences, Krakow PL-31342, Poland

**Keywords:** collagen, hyaluronic acid, hydrogels, hydrogel structure and rheology, bladder cancer

## Abstract

Collagen-hyaluronic acid (Col-HA) hydrogels are widely studied as biomimetic materials that recapitulate the environmental physical and mechanical properties crucial for understanding the cell behavior during cancer invasion and progression. Our research focused on Col-HA hydrogels as an environment to study the invasion of bladder cancer cells through the bladder wall. The bladder is a heterogeneous structure composed of three main layers: urothelium (the softest), lamina propria (the stiffest), and the muscle outer layer, with elastic properties lying between the two. Thus, the bladder cancer cells migrate through the mechanically distinct environments. We investigated the impact of Col-HA hydrogel microstructure and rheology on migrating bladder cancer T24 cells from the cancer spheroid surface to the surrounding environment formed from various collagen I and HA concentrations and chemical structures. The designed hydrogels showed variability in network density and rheological properties. The migration of bladder cancer cells was inhibited inside hydrogels of ∼1 kPa storage modulus. The correlation analysis showed that collagen concentration primarily defined the rheological properties of Col-HA hydrogels, but hydrogels can soften or stiffen depending on the type of HA used. Within soft Col-HA hydrogels, cells freely invade the surrounding environment, while its stiffening impedes cell movement and almost inhibits cell migration. Only individual, probably leading, cells are observed at the spheroid edges initiating the invasion. Our findings showed that the rheological properties of the hydrogels dominate in regulating cancer cell migration, providing a platform to study how bladder cancer cells migrate through the heterogeneous structure of the bladder wall.

## Graphical abstract

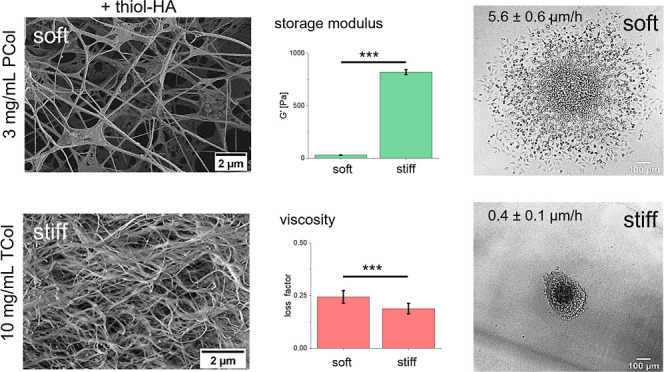

## 1 Introduction

The extracellular matrix (ECM) comprises a dynamic network of various elements surrounding the cells,^1,2^ strongly linked with the cell status and maintaining such processes as cell differentiation, proliferation, or migration needed for cell functioning.^3,4^ Collagen type I (Col I) is the most abundant ECM component, forming an-assembled fibrous network that alters depending on the cell state.^1,2,5^ Such remodeling can be a part of physiological processes like maintaining skin health^6^ or preventing bone loss.^7^ Collagen was found to be associated with hyaluronic acid (HA), the glycosaminoglycan distributed within the ECM of various tissues (e.g., skin, tendons, blood vessels, and connective tissues).^8^ HA molecules are highly hydrophilic, showing an extraordinary ability to retain up to 1000 times their weight in water.^9^ Consequently, depending on the tissue, HA serves numerous functions, e.g., it is known for its high hydrophilicity and is responsible for skin elasticity and aging.^10^ HA stabilizes ECM by connecting to collagen-fibrous networks.^11^ With cells, HA interacts with CD44 receptors responsible for, among others, adhesion and migration,^12,13^ hyaluronan degradation^13^ or lymphocyte activation.^14^ Another receptor for hyaluronan-mediated motility, i.e., RHAMM,^15^ is involved in the migration of fibroblasts based on protein tyrosine phosphorylation posttranslational modifications that are related to cytoskeleton and focal adhesion remodeling, the two processes affecting cell adhesion.^16^ It also impacts the transport of cytokines and growth factors responsible for tissue viscoelasticity throughout the tumor microenvironment. It alters tissue microstructure, mechanics, and perfusion.^17–19^

Hydrogels composed of collagen and HA mimic the ECM properties on structural and functional levels.^20^ In the native environment, collagen and HA create semi-interpenetrating networks.^21^ Physiologically, collagen molecules self-assemble into macroscopic hierarchical tissue matrices. The physical cross-linking of Col-HA hydrogels involves the aggregation of collagen molecules due to electrostatic and hydrophobic interactions to form fibrils, roughly cylindrical fibers reaching up to 500 nm in diameter and centimeters in length.^22^ Collagen matrices produced from atelocollagen, a form of collagen produced by enzyme extraction lacking in the nonhelical telopeptide region, show loosely tangled collagen fibers and form sponge-like matrices. In comparison, matrices formed from telocollagen, the acid-extracted form of collagen, form networks characterized by smaller pore sizes and increased mechanical properties.^23^ The large range of functional groups present in the structure of HA, i.e., hydroxyl, carboxyl, and acetyl groups, enables its chemical modifications, affecting the final physical properties of the hydrogels.^24^ Thiolated HA is the most common modification strategy for forming covalently cross-linked HA hydrogels,^25,26^ often performed by chemical reactions between ligands containing free thiol groups and the hydroxyl or carboxyl groups in HA molecules, inducing changes in the collagen 3D networks.^27^ HA can be associated with the surface of the collagen fibers^28^ or occupy the interstitial space between them,^29^ affecting the final structure and mechanics of the hydrogel.^28,30^ Collagen or collagen-hyaluronan (Col-HA) hydrogels have been widely studied for tissue engineering and regenerative medicine due to their biocompatibility, structural similarity to the extracellular matrix, and ability to support cell growth and differentiation.^27,29,30^

Pathophysiological processes like fibrosis or cancers are strongly linked with the alteration in the collagen network, in which its deposition is frequently observed.^31,32^ The increased^33^ or decreased^34^ Col I deposition can be associated with increased malignancy. Collagen contributes to tumor cell infiltration, expansion, and metastasis, especially collagen type I, which is strongly linked with cell growth, proliferation, differentiation, and inflammation.^35^ The presence of collagen in the proximity of tumors was seen as a stiff and fibrous barrier through which cancer cells disseminate.^36^ The regulation of the balance between tumor-inhibiting and promoting effects of macrophages,^37^ tumor angiogenesis,^38^ or the use of collagen fibers by cancer cells as tracks to escape from the primary tumor site^39^ are prerequisites for the predictive potential of collagens, especially for solid tumor, in which they are highly expressed.^35^ Malignant lesions are often stiffer than benign lesions due to the presence of connective tissue, where type I collagen is the main structural component.^27,40^ Combined with HA, it influences the transport of cytokines and growth factors responsible for tissue hydration and mechanics throughout the tumor microenvironment.^41,42^ Overproduction of HA in the tumor microenvironment often results in poor prognosis, the collapse of blood vessels, and inhibition of drug delivery.^43^ Therefore, key ECM components such as collagen or HA are the primary targets for studying the role of ECM in the progression of various cancers, including bladder cancers.^24,45,46^ The deposition of collagens in bladder cancer is no exception, as collagen I is a major component of the bladder wall.^44–46^ Its larger amount can promote the invasion of nonmuscle bladder cancer.^46–48^

Our study developed 3D Col-HA hydrogels with varied structures and mechanical properties using two forms of collagen I: telocollagen (native collagen, TCol) and atelocollagen (enzymatically processed telocollagen, PCol), which were combined with thiol-modified (thiol-HA) or fluorescein-amine-coupled hyaluronic acid (fHA). The resulting hydrogels served as a platform to study how bladder cancer cells (transitional cell carcinoma, T24 cells) migrate from spheroids to the hydrogels.

## 2 Materials and Methods

### 2.1 Reagents

PureCol (3 mg/mL, atelocollagen), PureCol EZ Gel (5 mg/mL, atelocollagen), FibriCol (10 mg/mL, atelocollagen), TeloCol (10 mg/mL, telocollagen) collagen type I bovine acidic solutions and Glycosil thiol-modified hyaluronic acid (thiol-HA) lyophilizate with a concentration of 10 mg/mL were purchased from Advanced BioMatrix (Cell Systems, Germany). Telo- and atelocollagen are referred to as TCol and PCol, respectively. Fluorescein (amino-fluorescein)-labeled hyaluronic acid (fHA), phosphate-buffered saline (PBS) tablets, fetal bovine serum (FBS), trypsin, and Live/Dead cell double staining kit were purchased from Merck (formerly Sigma-Aldrich, Poland). Roswell Park Memorial Institute Medium 1640 (RPMI 1640) cell culture medium was obtained from ATCC (LGC Standards, Poland). The sodium hydroxide micropellets and paraformaldehyde were bought in Avantor Performance Materials Poland. Collagen I monoclonal antibody (5D8-G9, 1 mg/mL) and Alexa Fluor 555 goat antimouse IgG (H + L) (2 mg/mL) were purchased from Invitrogen (ThermoFisher Scientific, Poland).

### 2.2 Collagen (Col) and Collagen-Hyaluronic (Col-HA) Hydrogel Preparation

Collagen hydrogels were prepared by mixing cold collagen solutions with 10 × PBS in an 8:1 v/v ratio. Next, the pH of the mixture was adjusted to 7.2–7.5 using 115 μL of 0.1 M NaOH solution in deionized water (dH_2_O). The sterile dH_2_O was added to the solutions to keep the final 8:1:1 v/v ratio. Col hydrogels were used alone and combined with thiol-HA and fHA, prepared in standard 24-well plates (Genos, Poland) by mixing thiol-HA diluted in 1 mL of 10 × PBS with dH_2_O to keep the 8:1:1 v/v ratio. Col-fHA hydrogels were prepared by mixing collagen solutions with 5 μg of fluorescein-labeled HA to obtain the final solution with a 200:1 v/v ratio. Col-fHA hydrogels were prepared in glass-bottom 24-well plates (Genos, Poland). All the procedures were carried out on ice to prevent gelation while preparing hydrogels. In the next step, samples were incubated at 37 °C in an atmosphere of 5% CO_2_ for 120 min to induce polymerization in a whole hydrogel volume.

### 2.3 Cell Culture and Spheroid Formation

Bladder cancer T24 cells (ATCC, being accessible at the cell bank located at the Department of Biophysical Microstructures, Institute of Nuclear Physics, Polish Academy of Sciences, Kraków, Poland) were cultured in tissue culture flasks 25 cm^2^ (TPP, Genos, Poland) containing RPMI 1640 culture medium ATCC (LGC Standards, Poland) supplemented with 10% FBS (Merck, Poland), which is referred here as to complete culture medium. No antibiotics were used for the cell cultures. Cells were grown in a CO_2_ incubator (Nuaire), providing an optimal culture condition, i.e., 37 °C, 5% CO_2_, and 95% humidity.

T24 cells were used to form bladder cancer spheroids in the following way. Cells were centrifuged after trypsinization with 0.1% trypsin–EDTA solution (Merck, Poland) (1800 rpm, 4 min), the supernatant was removed, and cells were suspended in the complete culture medium. Then, they were counted using the Burker chamber. Spheroids were cultured in 96-well U-bottom 3D plates (ThermoFisher Scientific, Poland), where 3000 cells/well were seeded and cultured in RPMI 1640 media supplemented with 10% FBS in a CO_2_ incubator. After 3 days of culture, spheroids were collected from the U-bottom plates, placed on the top of an unpolymerized solution of collagen hydrogels (1 mL) in the 24-well plates, and left to immerse freely into hydrogels. Next, they were moved to a CO_2_ incubator for polymerization (37 °C, 5% CO_2_, 120 min).

### 2.4 Migration of Bladder Cancer T24 Cells in 3D Col-HA Hydrogels

To observe the migration of cells escaping from the spheroids, spheroids cultured in 96-well U-bottom 3D plates were collected from the plates and placed on top of an unpolymerized solution of collagen hydrogels in the 24-well plates. Next, 24-well plates were moved to the CO_2_ incubator for polymerization (37̊°C, 5% CO_2_, 120 min). Afterward, the wells were filled with 1 mL of complete culture medium, and the spheroid growth, conducted in the CO_2_ incubator, was observed after 24 h, 48 h, and 72 h. Spheroids were located at various depths inside the Col-HA hydrogels (thickness of 2 mm). After each time, phase-contrast images of spheroids were acquired using an optical microscope (Olympus IX83) with 4× (UPLAN FLN 4×/0.13) 10× (UPL FLN2 10×/0.3) objectives and the CMOS camera type Prime BSI 166 Express Scientific (01-prime-BSI-EXP, Photometrics). Images of the individual spheroid were recorded for each hydrogel, and they were used to quantify the migration distance (expressed as a mean ± standard deviation for *n* = 15 spheroids). The migration distance was determined using CellSens Dimension software (Olympus) as follows. First, the core of the spheroid was marked. Next, the position of each migrating cell beyond an average migration distance was quantified as the length of a linear segment connecting the surface of spheroids and cells located outside the marked area (graphically presented in Supporting lnformation. Figure S4). The data are presented as a mean ± standard deviation from 3 biological repetitions.

### 2.5 Scanning Electron Microscopy

The 1000 μL of hydrogel solution was transferred into 24-well plates, polymerized as described above, and fixed with 0.5% glutaraldehyde solution (Avantor Performance Materials Poland), followed by rinsing in _d_H_2_O. Next, they were dehydrated by freezing them with liquid nitrogen and lyophilized in the chamber under a controlled pressure of 10^–5^ Pa for 12 h. Such freeze-dried samples were mounted with double-sided carbon tape onto SEM holders and coated with a 5 nm gold layer using rotary-pumped sputter coating (Q150RS, Quorum Technologies, UK). Hydrogel microstructure was imaged using a scanning electron microscope (Merlin Gemini II, Zeiss, Germany), working at a current of 90 pA and voltage of 5 kV. ImageJ software (https://imagej.net, version 1.54p) quantified the fiber diameter and pore size (long-axis) from 200 analyzed fibers (or pores) for each hydrogel type. Because of a strong intertwined network, only the small pores were considered.

### 2.6 Fluorescent Labeling of Collagen and Hyaluronic Acid

160 μL of the prepared Col-fHA solution was transferred to 24 well glass-bottom microplates (Greiner Bio-One, Merck, Poland) and incubated at 37 °C in an atmosphere of 5% CO_2_ for 60 min. After polymerization, samples were incubated in PBS at 37 °C for 24 h. Next, samples were incubated with 1 μg/mL collagen I monoclonal antibody for 6 h at 25 °C. After rinsing them five times in PBS for 30 min, they were incubated with 2 μg/mL Alexa Fluor 555 goat antimouse IgG secondary antibody for 6 h at 25 °C. Fluorescence images were captured by an inverted microscope Olympus IX83 (Olympus, Japan) with an objective 40× (LUC PLAN FLN 40×/0.60). This microscope acquired fluorescent images using the CMOS camera type Prime BSI 166 Express Scientific (01-prime-BSI-EXP, Photometrics). The recorded image size was 2048 px × 2048 px, with a resolution of 325 nm/px). A 100W mercury lamp was applied to excite fluorescent dyes.

A U-FBW filter (λ_ex_ = 460–495 nm, λ_emi_ = 510 nm) was used to collect fluorescent images of HA (amino-fluorescein), while a U-FGW filter (λ_ex_ = 530–550 nm, λ_emi_ = 575 nm) was applied to collect fluorescently labeled collagen fibers (Alexa Fluor 555).

### 2.7 Co-Localization Analysis

Co-localization analysis was obtained using the ImageJ software (https://imagej.net) with a supplemented Just Another Co-localization Plugin (JaCoP plugin,^49^ https://imagej.net/plugins/jacop) for individual fluorescence images acquired for each collagen hydrogel type (two channels were recorded, i.e., collagen (red, *n* = 5 images) and fHA (green, *n* = 5 the corresponding images). The relationship between the signal intensities in the two images was assessed using Pearson’s coefficient calculated by linear regression, providing the association rate of the two dyes (denoting a colocalization percentage). Additionally, the analysis of Manders’ coefficients^50^ was conducted to evaluate to what extent the overlapping coefficients M1 (green signal overlapping red signals) and M2 (red signal overlapping green signal) are the indicators of to what extent the green signal coincident with a signal in the red channel (or the opposite). The Costes’ algorithm was applied because it iteratively searches for a threshold where the Pearson correlation coefficient reduces to zero for all pixels below the intensity threshold set to separate background noise from signals.^51^

### 2.8 Rheology of Hydrogels

For rheological measurements of collagen hydrogels, 1 mL of hydrogel solutions was transferred into 24-well plates and incubated for 120 min in a CO_2_ incubator at 37 °C. After polymerization, samples were incubated with PBS at 37 °C for 24 h. Afterward, individual hydrogel samples were cut from plates with a custom-made copper tube with a diameter of 8 mm corresponding to the diameter of a parallel plate geometry used in rheological measurements. The thickness of the sample was kept constant at 2 mm. A liquid container was filled with PBS, and additional covering was used to avoid evaporation. In a rheometer (MRC302, Anton Paar, Graz, Austria), we used sandblasted parallel plates (8 mm in diameter) to avoid hydrogels sliding. We applied amplitude sweep measurements at a frequency of 0.1 Hz^52^ and shear strains 0.01–100%, which we used to determine the linear viscoelastic range (LVER), where the mechanical response of the sample is independent of the applied amplitude. The results enabled us to obtain the maximum strain value we can conduct, while the lack of amplitude dependency denotes that the sample behaves linearly. Within LVER, *G*′ and *G*″ are independent of deformation.^53^ LVER was determined by fitting a linear function to the parallel region in the *G*′/*G*″– shear strain curves. The storage (*G′*′) and loss (*G*″) moduli were determined as follows.^54^ The strain γ applied to the material subjected to small oscillations is described as γ = *A*·sin·(ω·t), where *A* is the amplitude and ω is the frequency of the oscillations. For purely elastic materials, the stress required to impose deformation is proportional to the strain, while for viscoelastic liquids, the stress is proportional to the shear strain rate, i.e., γ̇ = A·ω·cos­(ω·t). For viscoelastic materials like collagen hydrogels, the response is placed in between, and it is described by the complex shear modulus G* = G′*+ i*·*G*″, having two contributions, i.e., in-phase corresponding to *G*′ and out-of-phase related to *G*″*.* The phase shift δ is always between 0°to 90°, thus $G^{\prime }=\frac{stress}{strain}\cdot \cos\left(\delta \right)$ , while $G^{\prime \prime }=\frac{stress}{strain}\cdot \sin\left(\delta \right)$ . In our work, both moduli were plotted as a function of shear strain. They are the elastic and viscous components of the hydrogel sample, respectively. Depending on the Col-HA type, 6 or 9 hydrogel samples were measured.

### 2.9 Statistical Analysis

The mean value and the corresponding standard deviation (shown as error bars on each plot) were calculated for all results from *n* samples, cells, or images. Statistical analysis was conducted using OriginPro 2022 software. Statistical significance for pore and fibril diameters, rheological results, and cell migration was assessed using a one-way ANOVA at α = 0.05. An unpaired Student’s *t*-test at α = 0.05 was applied for colocalization. Notation: *p* < 0.05 (*), *p* < 0.01 (**), and *p* < 0.001 (***).

## 3 Results

### 3.1 Col-HA Hydrogel Structure Depends on Collagen and HA Types

In our study, we prepared 3D hydrogels from atelocollagen type I (PCol, collagen concentration of 3 mg/mL) and telocollagen (TCol, 10 mg/mL). TCol contains triple helical and nonhelical regions, while PCol is composed of analogous collagen molecules, but nonhelical regions are truncated (Figure 1A). The collagen solutions were mixed either with thiol-modified HA (thiol-HA) or with fluorescently labeled HA, where carboxyl groups are replaced with fluorescein (fHA), followed by their polymerization. We investigated the Col-HA hydrogel microstructure using scanning electron microscopy (SEM, Figure 1B,C).

**Figure 1:**
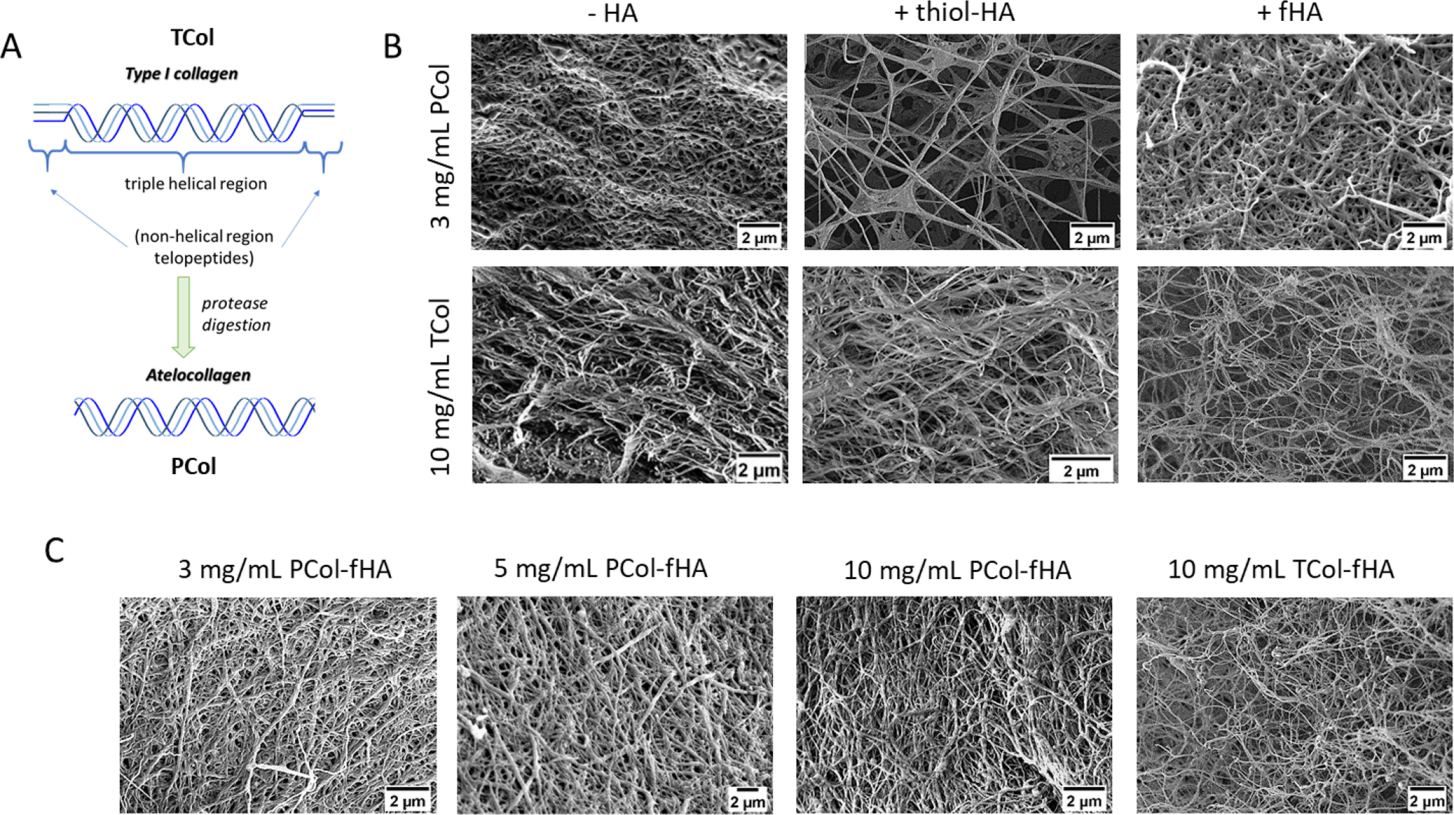
Microstructure of collagen-based (Col-HA) hydrogels. (A) schematic explanation of the structural differences between atelocollagen type I (PCol) and telocollagen (TCol). (B) SEM images of collagen hydrogels without and with added HA (either thiol-modified or fluorescently labeled). (C) Analogous SEM images for collagen hydrogels with fHA, prepared for 3, 5, and 10 mg/mL PCol concentrations. The results are compared to the structure of 10 mg/mL of TCol-based hydrogels.

The images of all hydrogels showed different morphological structures with large pores intertwined with the small ones. The morphometric parameters, such as the size of the small pores and fibril diameters, were determined from the acquired SEM images (Supporting Information. Figure S1). PCol (3 mg/mL) and TCol (10 mg/mL) hydrogels without HA showed a distinct network of collagen fibers characterized by smaller fiber diameter and larger pore size (*p* < 0.001) in the case of TCol-based hydrogel (morphometric analysis is included in Supporting Information. Table S1). Adding HA changed the hydrogel’s structure depending on the HA. For PCol (3 mg/mL), adding thiol-HA resulted in a massive increase in the pore size (2.03 ± 1.35 μm) as compared to TCol (10 mg/mL), while the fibril diameter was preserved. Notably, the standard deviation for both quantities is about 50–70%, indicating considerable heterogeneity inside the hydrogel. Using fHA, where the carboxyl group is replaced by amino-fluorescein to make HA fluorescent, did not affect the pore size, but the fibril diameter was significantly lower than in PCol (3 mg/mL). The presence of thiol-HA in TCol (10 mg/mL) increased both the fibril diameter (*p* < 0.001) and the pore size (*p* < 0.001). A similar effect was observed when fHA was applied to produce Col-HA hydrogels, for which larger fibril diameter (*p* < 0.001) and pore size (*p* < 0.001) were noted. In the next step, we studied how atelocollagen (PCol) concentration in the Col-fHA hydrogels affects the pore size and fibril diameter. An increased concentration, i.e., 5 mg/mL PCol, induced an increase in the pore size (*p* < 0.0001) and fibril diameter (*p* < 0.0001). Further increase of the PCol concentration up to 10 mg/mL did not affect the pore size. However, we still observed an increase in fibril diameter (*p* < 0.0001). In conclusion, the microstructure of Col-HA hydrogels depends on the collagen type and concentration used. The effect of HA on hydrogel structure was entirely different and dependent on the type of HA.

### 3.2 HA Aligns Mostly along the Collagen Fibers

Various morphological images for Col-HA hydrogels, especially the HA type diverse effect on the hydrogel structure observed in our study, ask for an evaluation of how collagen fibers and HA align. A complete and partial alignment of HA with collagen fibers could be expected (Figure 2A), especially in the case of two different collagens used in our study (PCol or TCol) and distinct levels of HA modifications. Therefore, in the next step, we fluorescently labeled collagen I and applied HA coupled with amino-fluorescein molecules (Figure 2B).

**Figure 2:**
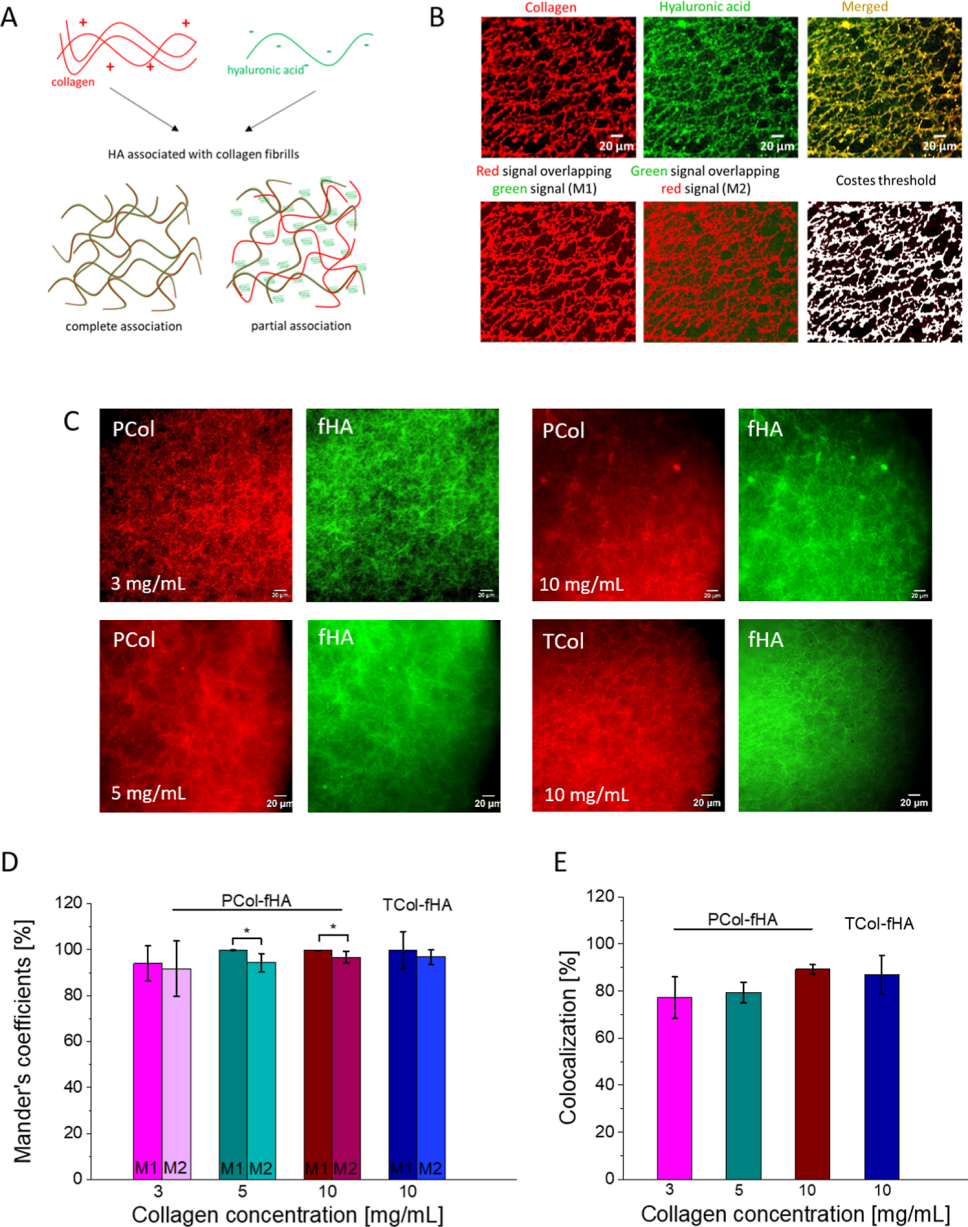
HA alignment along collagen fibers verified for atelo- (PCol) and telocollagen (TCol). (A) schematic explanation of the potential misalignment between HA and collagen fibers. (B) The workflow used for colocalization analysis. The upper panel shows collagen labeled with Alexa Fluor 555 (red), HA with amino-fluorescein (green), and a merged image. The bottom panel presents overlaid images (green over red signals and red over green signals) and the resulting Costes threshold used to separate background noise from the fluorescent signal. (C) Representative fluorescent images of all groups of Col-HA hydrogels studied (separate images of collagen and HA). (D) Manders’ coefficients indicate how the red over green (M1) and green over red (M2) signals overlap, affecting the determination of the actual degree of correlation between these signals (expressed as mean ± standard deviation from *n* = 5 images). Statistical significance was obtained using an unpaired Student’s *t*-test at α = 0.05. (E) Comparison of colocalization degree in Col-HA hydrogels with varying collagen concentration and same concentration of fHA, expressed as mean ± standard deviation form *n* = 5 images.

Two parameters, i.e., Manders’ coefficients and colocalization percentage, were calculated to quantify the association between fluorescently labeled collagen and HA. The Costes’ threshold was applied to separate background noise from the fluorescent signals. The obtained Mander’s coefficients M1 (red over green signals) and M2 (green over red signals) were of a similar order of 91–99% (Figure 2C). The overall colocalization percentage was 77.3 ± 8.7% for 3 mg/mL PCol, 79.3 ± 4.3% for 5 mg/mL PCol, 89.2 ± 2.1% for 10 mg/mL PCol, and 86.9 ± 8.3% for 10 mg/mL TCol (Figure 2D). The results showed that fHA and collagen colocalization percentage for the studied Col-HA hydrogels was around 80%–90%, indicating that HA tends to bind or align along the collagen fibers. Only 10–20% of HA is freely located in the space between collagen fibers. Moreover, the higher colocalization percentage observed for larger collagen concentrations indicates better alignment between HA and collagen. These results demonstrate that the HA aligns mostly along the collagen fibers. The elaborated and presented protocol for Col-HA hydrogel fabrication results in hydrogels characterized by high alignment between collagen fibers and hyaluronic acid.

### 3.3 Rheology of Col-HA Hydrogels is Affected by HA

Next, we asked ourselves how the rheological properties of Col-HA hydrogels depend on collagen and HA types. We used a plane–plane geometry in the oscillatory rheometer to measure the rheological properties of Col-HA hydrogels in a designed chamber, allowing for a liquid environment to prevent the drying of hydrogels (Figure 3A). The amplitude sweep enabled the determination of storage *G*′ and loss *G*″ moduli as described in Materials and Methods (the relations between *G′* or *G*″ and strain rate, recorded at 0.1 Hz, from which the storage and loss moduli are presented in Supporting Information. Figure S2).

**Figure 3:**
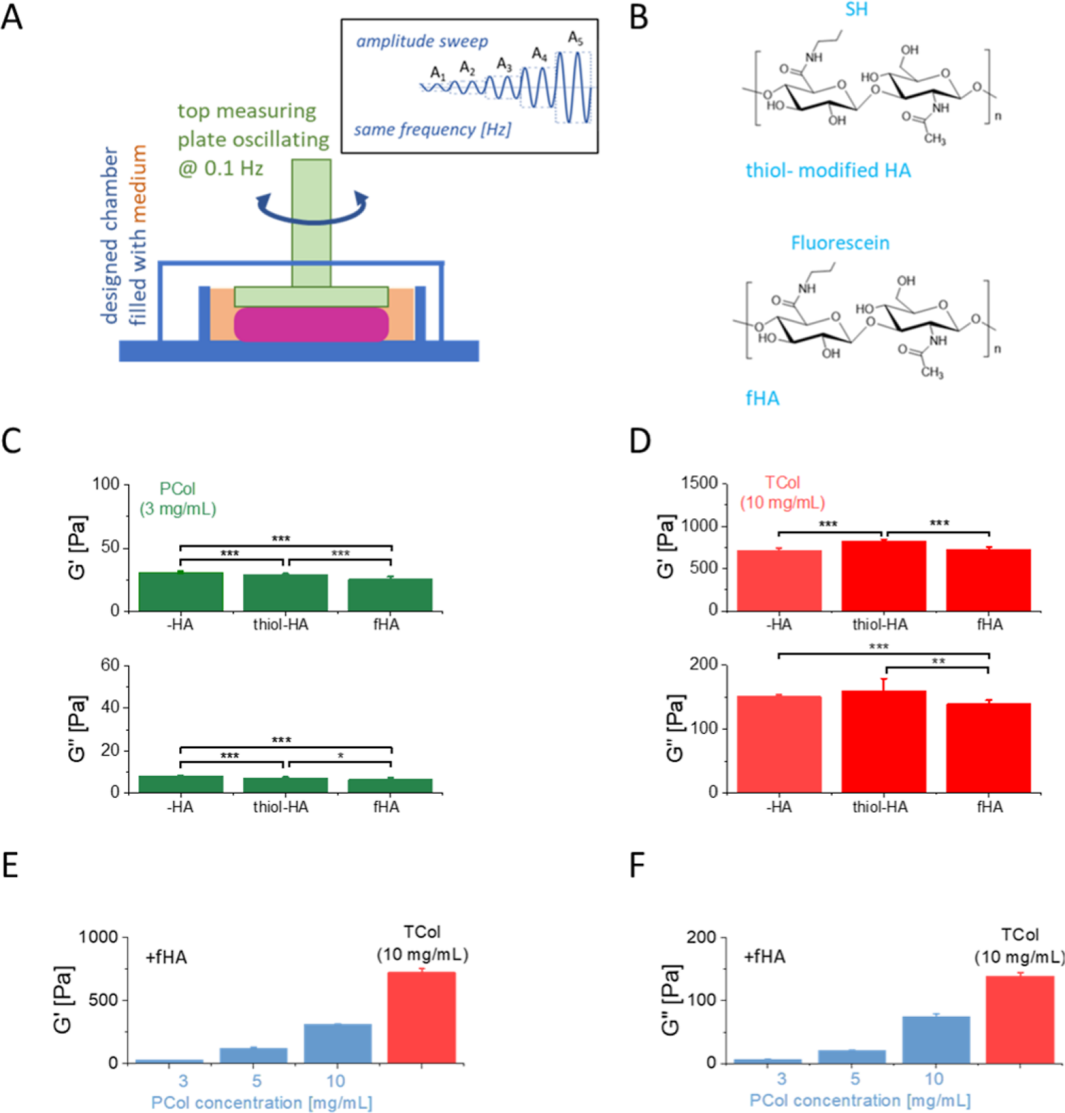
Rheological properties of Col-HA hydrogels. (A) schematic presentation of the experimental setup and amplitude sweep measurements. (B) Structural differences among the HA used for the experiments in which amino-fluorescein replaced the thiol group. (C–F) Comparison of storage and loss moduli determined from the LVER regions for all studied groups of hydrogels. Data are presented as a mean ± standard deviation from 6 to 16 points included in the LVER region. Statistical significance was quantified using a one-way ANOVA at α = 0.05.

First, we studied how rheological properties altered depending on HA modification with two types of HA (Figure 3B–D). Next, changes in rheology were evaluated as a function of PCol concentration (3, 5, 10 mg/mL) in Co-HA hydrogels (Figure 3E–F). For hydrogels composed of 3 mg/mL PCol, HA softens the produced hydrogels, i.e., storage and loss modulus decreased by about 10–25% (Figure 3C). For hydrogels composed of 10 mg/mL TCol, the storage and loss modulus changes were also in the same range, i.e., ± (14–25) %, leading to either stiffening for thiol-modified HA or softening for fHA hydrogels (Figure 3D). The rheological properties of Col-HA hydrogels were measured as a function of PCol concentration, and compared to 10 mg/mL TCol, they showed a concentration-dependent increase (Figure 3E–F). Notably, the Col-HA hydrogels containing 10 mg/mL of PCol was still softer than hydrogels produced using 10 mg/mL of TCol, i.e., *G*′ = 309.3 ± 5.9 Pa (*n* = 6 points within LVER region) versus *G*′ = 721.1 ± 30.4 Pa (*n* = 13) and *G*″ = 74.1 ± 4.5 Pa (*n* = 16) versus *G*″ = 139.2 ± 5.6 Pa (*n =* 16), respectively. Alongside storage modulus changes, the loss tangent (defined as a ratio *G*″ to *G*′,) changed depending on the Col-HA hydrogel type, ranging between 0.16 and 0.25 (Table 1).

**Table 1:** Rheological Parameters Obtained for the Studied Col-HA Hydrogels with *p*-values Determined Relative to 3 mg/mL PCol Combined with Thiol-HA or fHA

Col-HA type	*G*′ [Pa]	*G*″ [Pa]	*G*″/*G*′	*p*-value
3 mg/mL PCol + thiol-HA	28.83 ± 1.12	7.03 ± 0.60	0.244 ± 0.03^a^	
10 mg/mL TCol + thiol-HA	821.86 ± 23.33	154.46 ± 16.06	0.188 ± 0.025	0.006^b^
3 mg/mL PCol + fHA	25.27 ± 2.12	6.29 ± 0.99	0.249 ± 0.06	
5 mg/mL PCol + fHA	121.93 ± 5.90	20.23 ± 0.72	0.166 ± 0.014	0.008
10 mg/mL PCol + fHA	309.33 ± 5.88	74.08 ± 4.53	0.239 ± 0.019	0.705
10 mg/mL TCol + fHA	721.05 ± 30.35	139.21 ± 5.63	0.193 ± 0.016	0.516

a Errors were determined as maximal errors.

b in relation to 3 mg/mL PCol and respectively HA.

Col-HA hydrogels’ rheological properties depend on the collagen and HA types. HA (regardless of the type) softens the pure collagen matrix of the same concentration, but the viscous contribution remains within the range of 16%–25%. When the concentration of collagen increases, the storage and loss moduli increase while preserving a similar level of viscous component. The atelocollagen was more sensitive to the type of HA, which caused either stiffening (thiol-modified HA) or softening (fluorescein-modified HA), keeping a viscous contribution of ∼19%, i.e., between the range observed for pure collagen. The stiffest Col-HA hydrogels were obtained for 10 mg/mL TCol with thiol-modified HA.

### 3.4 Bladder Cancer T24 Cell Migration Strongly Depends on Rheology and Composition of Col-HA Hydrogels

The fabricated Col-HA hydrogels were used as a platform to evaluate the invasion of cancer cells to TME. We tested the capability of bladder cancer T24 cells to migrate into prepared Col-HA hydrogels, as these cells have already been shown to be highly migrative and invasive.^55^ Here, we study time-dependent migration to Col-HA hydrogels composed of various collagen concentrations and types (PCol, TCol, viability results are presented as Supporting Information. Figure S3) by quantification of the maximum distance *D* reached by single cells escaping from the spheroid (Supporting Information. Figure S3, Supporting Information. Table S2). The results showed that soft hydrogels are more suitable for bladder cancer T24 cell migration than stiff hydrogels (Figure 4).

**Figure 4:**
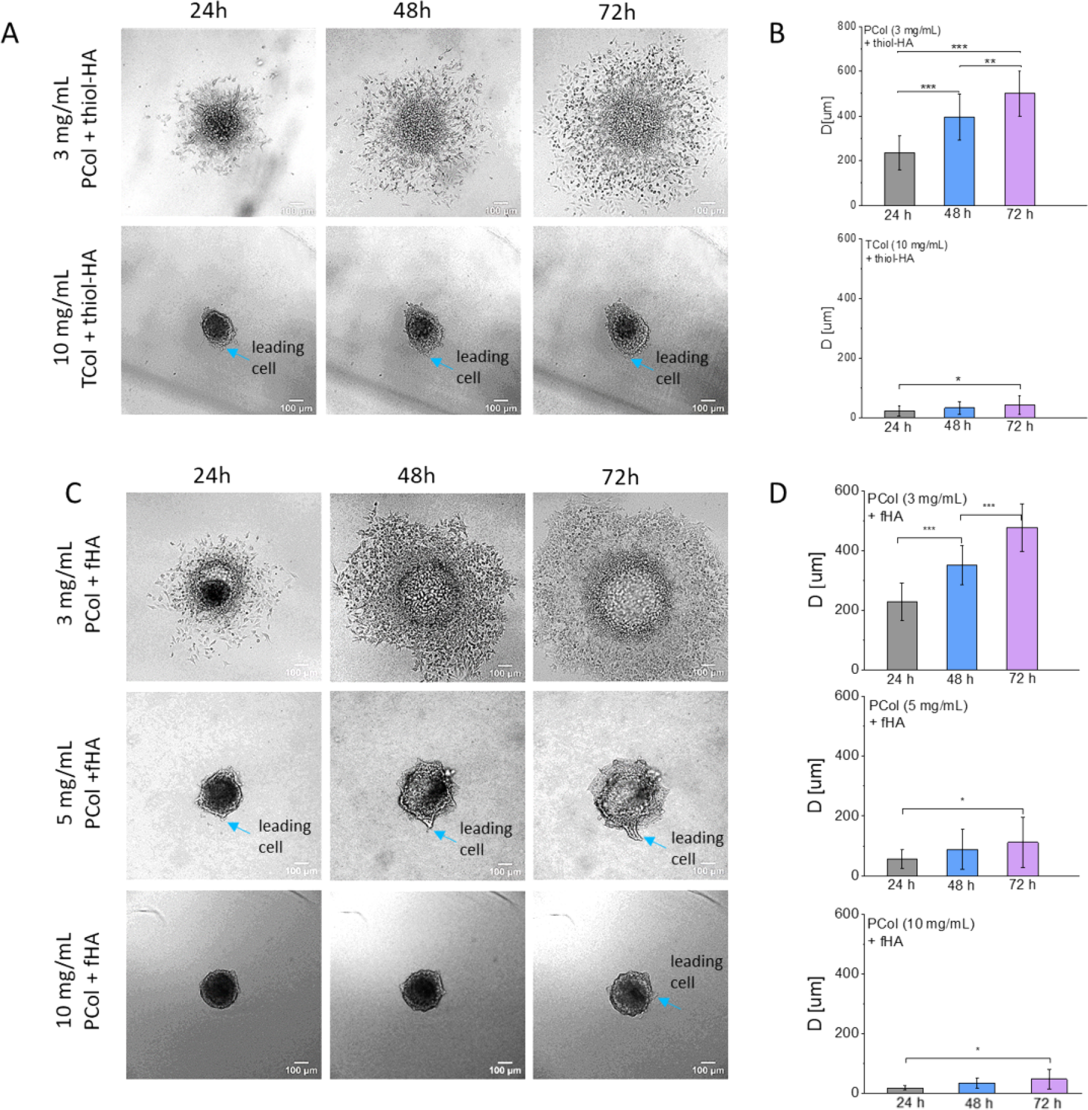
Migration of bladder cancer T24 cells into 3D Col-HA hydrogels. (A) The migration of T24 cells inside PCol (3 mg/mL) + thiol-HA and TCol (10 mg/mL) + thiol-HA hydrogels. (C) The migration of cells into PCol (3 mg/mL) + fHA hydrogels was studied as a function of PCol concentration. Arrows indicate a leading cell initiating cell migration. (B,D) The corresponding time-dependent migration distance. Data are presented as a mean ± standard deviation from 3 biological repetitions. Statistical significance was quantified using a one-way ANOVA at α = 0.05.

In the softest Col-HA hydrogels containing 3 mg/mL PCol, almost all cells escape from the spheroid surface, regardless of the HA used, i.e., thiol-HA or fHA. Increasing the collagen concentration using both PCol and TCol resulted in lower migration of T24 cells. Instead of migration of all cells, for some cases, only the single protrusions are observed (indicated by arrows, Figure 4). They are more present in Col-HA hydrogels formed from 5 mg/mL PCol at all time spots, and they are barely visible for Col-HA hydrogels composed of 10 mg/mL PCol or 10 mg/mL TCol, in which few protrusions are observed after 72 h. Their triangular shape suggests that at the apex, an individual cell guides the collective migration of bladder cancer cells. In conclusion, the largest migration of these cells is observed for the softest Col-HA hydrogels, composed of 3 mg/mL PCol, regardless of the HA type used. Increasing PCol concentration or replacing it with TCol strongly limits cell migration.

## 4 Discussion

Challenges persist in producing reproducible Col-HA hydrogels with consistent properties. Variations in mechanical strength, swelling behavior, and cross-linking significantly impact their performance in clinical applications.^30,56,57^ Our primary goal was to develop a simplified model of Col-HA hydrogels that could be used to study the migration of cancer cells. Since the migration of cells is a complex phenomenon involving distinct biological and physicochemical processes,^58–60^ we begin with the structural and rheological properties of Col-HA hydrogels.

Achieving consistent cross-linking throughout the hydrogel volume, especially when HA is incorporated, can be challenging and leads to heterogeneity in physical and biological properties.^61,62^ Adding HA helps create a hydrated environment crucial for cell viability.^63^ Therefore, maintaining an appropriate balance between hydration and mechanical integrity can be difficult, particularly for cellular applications where moisture levels must be controlled and maintained to keep cells alive. To produce a broad range of Col-HA hydrogels, two types of collagen I were applied, i.e., telo- and atelocollagen, in which helical regions were removed by protease digestion. Hydrogel solutions were incubated at 37 °C to maintain constant polymerization kinetics and form homogeneous samples. The structure of Col-HA hydrogels showed randomly oriented fibers with varying pore sizes and fiber diameters dependent on the collagen and HA types used to produce them. The structure shows larger pores with smaller pores intertwined. The fiber diameter ranged from 60 to 102 nm, while the size of the smallest pores varied between 200 and 250 nm, except for Col-HA hydrogel composed of 3 mg/mL PCol and thiol-modified HA. In this case, the diameter of the small pores reached 2.03 ± 1.36 μm. Since the same collagen type was applied to produce hydrogels supplemented with two types of HA (i.e., thiol- and fluorescein-modified HA, respectively), the significant increase in pore size was attributed to the thiol modifications of HA. This effect was also observed for 10 mg/mL TCol, but the change was not as notable as for PCol-based hydrogels. We hypothesized that differences in the alignment of HA with collagen fibers are responsible for the larger pore size. Knowing the degree of Col I and HA alignment is essential because, in the native environment, cells interact with HA using CD44 membrane receptors.^64^ Thus, the distribution of HA is critical in cell adhesion and migration. Previously, the fluorescein-modified HA was used to localize the HA in Col-HA hydrogels, showing that in vitro HA exists both colocalized with collagen fibers and in the interfibrillar spaces, affecting the equilibrium and nonequilibrium mechanical properties of hydrogels.^65^ However, the colocalization analysis showed a high association of fHA with atelo- and telocollagen-formed hydrogels (77% to 95%), indicating that HA aligns mostly along the collagen fibers. It has already been reported that pore size, fiber diameter, and elastic modulus increase with HA concentration.^43^ According to manufacturer data, the thiol-HA used in our experiments falls between 0.55 and 0.75 μmol/mg, resulting in a thiolation degree between 20 and 30%, obtained using the Ellman’s test.^66^ (https://advancedbiomatrix.com/glycosil.html). The fHA is characterized by a 4–8 mol % substitution degree. Thus, the concentration of thiol-HA inside the Col-HA hydrogels was larger than in the case of using fHA; however, since there was no clear correlation between pore size (fiber diameter) and HA concentration for our hydrogels (Figure 1), we cannot explain larger pore size for 3 mg/mL PCol-thiol-HA hydrogels. Next, we consider that the highly hydrophilic HA molecules control the swelling ratio, leading to increased pore size concentration.^67^ In our case, Col-HA hydrogels were handled in liquid conditions, minimizing the swelling effect. Therefore, the large pore diameter can be explained by the thiolation effect rather than the location of HA in the collagen network. However, the hydrogel structure is also controlled by electrostatic interactions between charged functional groups in Col I and HA;^68^ thus, slight changes in pH might also contribute to the final structure of the hydrogels.

Designing and fabricating hydrogels of a similar order of mechanical and/or rheological properties resembling the physiological tumor environment is paramount. The physical features of the TME are known to regulate and correlate with cancer cell migration and invasion. The hydrogel mechanics influence cell fate,^69–71^ which is linked to the fact that all biological tissues and cells are viscoelastic,^72–74^ a phenomenon allowing them to behave differently depending on the external forces from being elastic for short-term responses to being viscous for longer periods.^75–77^ Our results showed that Col-HA hydrogels formed from atelocollagen are more compliant than hydrogels containing telocollagen (2.5 fold even if the same concentration of both collagen types was used). Reciprocity along the line spheroid surface and microenvironment asked for the rheological properties of hydrogels, which for our hydrogels were dependent on the specific types of Col I and HA ranging from 25 to 820 Pa. The viscous contribution remained similar, i.e., ∼16%–25%, as presented in Table 1. In most healthy biological tissues, the ratio between elastic and viscous contributions varied between 10% and 25%.^74,78–81^

Spheroids, a common 3D in vitro tumor model,^82^ are used to study cell migration and cancer invasion.^83^ For this purpose, spheroids must be incorporated into the functional biomaterials, providing the microenvironment with adjustable microstructure, chemistry, and mechanics similar to the native TME to facilitate their movement.^43^ The migration mechanisms involve the mechanics of cancer cells and the structural and rheological properties of the surrounding microenvironment, including cross-linking density, pore size, or collagen fiber orientation.^84^ In our case, we applied fabricated Col-HA hydrogels to evaluate the migration of bladder cancer T24 cells since these cells display high invasion and migration potential. Our results showed that the rheological properties of TME can promote and suppress cell migration inside hydrogels. The process is stiffness-dependent. In very soft hydrogels, cells migrate enormously, reaching large distances of ∼500 μm after a 3 day culture (Supporting Information. Table S2). Stiffening of the Col-HA hydrogels limits the cell motility, which is visible as a smaller migration distance and significantly lower number of migrating cells. Initially, the size of the small pores was considered since it may prevent cell migration when being of nm size and allow it for micron-scale ones for highly deformable bladder cancer T24 cells.^85,86^ However, there was no correlation between the migration and morphology of hydrogels (Supporting Information. Figure S4, Suppl. Note. Spearman correlation coefficient). By preparing dense Col-HA hydrogels with small pore sizes, we believe to limit the migration mechanisms to cell deformability and remodeling of the Col-HA network. Therefore, the migration distance, determined after 72 h of culture, was strongly associated with rheological properties of Col-HA hydrogels, i.e., with storage and loss moduli (−0.900, Spearman correlation coefficient), thereby, with collagen concentration (−0.949, Spearman correlation coefficient), suggesting that bladder cancer cell migration depends on the rheological properties of the tumor microenvironment. The successful invasion relies on the ability of cancer cells to migrate throughout the TME. It has already been reported that T24 cells are much softer than normal reference cells.^71,86^ This allows them to squeeze when passing through the pores of the Col-HA network. When pores are too small, then by active attachments of the cytoskeleton through the plasma membrane to the TME, they generate forces that pull attachments rearward and the cell forward^87^ and, by this form, tracks with larger pore size. These forces result from the balance between stress accumulated locally at the attachment points and forces generated by cells needed to overcome the stress. The positive net value allows cells to move, while values close to zero will inhibit cell migration. We observed this phenomenon for T24 cells in Col-HA hydrogels, more pronouncedly in hydrogels with storage modulus above 100 Pa. Observed individual protrusions still indicate that some cells can resist the mechanical stress generated in this environment. We could probably observe enhanced migration of these cells with longer culture time. The stiffer environment (storage modulus of ∼1 kPa) inhibits the migration of cancer cells as even no leading cells are present; however, the spheroid diameter increases with weakly visible individual cells trying to escape from the spheroid. These cells can get additional forces from expanding the spheroid and use pre-existing microtracks formed by collagen fibers as natural pathways for their movement.^35,36^ Our findings are important to understand how bladder cancer cells migrate through the bladder wall, a structure composed of structural layers characterized by distinct mechanical properties. Vidal et al.^88^ demonstrated that the healthy bladder wall is a mechanically inhomogeneous tissue. Its stiffness (described by elastic (Young’s) modulus, measured by atomic force microscopy (AFM) and nanoindenter) increases from the urothelium to the lamina propria, followed by a large decrease in the muscle outer layer. The results collected for murine bladder cancer showed urothelium as the softest layer, while the elastic modulus of the lamina propria and the muscle outer layer in cancer did not differ much. Notably, the neoplastic microenvironment was softer than that of healthy tissue, even if the physiological aging of these animals accompanied it. Taking into account the migratory properties of the softest bladder cancer cells,^86^ we showed that the stiffness of the environment of bladder cancer cells that is too large can inhibit migration.

## 5 Conclusions

In summary, we presented the protocol for manufacturing Col-HA hydrogels with varying physical and chemical properties, reproducible, and well-established structures (fiber diameter, pore size, and colocalization between Col and HA). The collagen concentration has mostly defined the rheological properties of Col-HA hydrogels, but hydrogels can soften or stiffen depending on the type of HA used. The increased collagen concentration leads to stiffening of the hydrogels, simultaneously inhibiting the migration of bladder cancer cells. We demonstrated the importance of a better understanding of the role of structural and rheological properties of 3D collagen-based hydrogels on cancer cell invasion into the surrounding microenvironment. This study demonstrates that combining cancer spheroids with a well-designed functional material—exhibiting specific chemical, biological, structural, and mechanical properties—offers a promising platform for exploring the effects of microenvironmental heterogeneity of the bladder wall on bladder cancer progression.

## Supplementary Material



## Notes

Morphometric analysis of Col-HA hydrogels (Table S1, Suppl. Figure S1); Rheological properties of Col-HA hydrogels (relations between storage and loss moduli versus shear strain, Suppl. Figure S2); Validity of bladder cancer cells in spheroids (MTS results, Suppl. Figure S3); Migration of bladder cancer cells inside 3D Col-HA hydrogels (Suppl. Figure S4, Suppl. Table S2). Comparing thiol-HA and fHA; Spearman correlation coefficient (Suppl. Table S3). (PDF)
