# Supplementary material for: Structural and Mechanical Characterization of Collagen-Hyaluronan Hydrogels Used to Study Cancer Cell Invasion through the Bladder Wall
Source: ACS Biomater Sci Eng. 2025 May 15;11(6):3443–54. doi: 10.1021/acsbiomaterials.5c00136 (PMC12152842; doi:10.1021/acsbiomaterials.5c00136)
Supplement: Supplementary file 1 [file ab5c00136_si_001.pdf]

Supplementary materials for

Structural and mechanical characterization of collagen-hyaluronan hydrogels used to study cancer cell invasion through the bladder wall

Sara Metwally[1], Justyna Śmiałek-Bartyzel[1], Joanna Pabijan[1], Małgorzata Lekka[1,]*

1 Institute of Nuclear Physics, Polish Academy of Sciences, PL-31342 Krakow, Poland

*Corresponding authors:

malgorzata.lekka@ifj.edu.pl, sara.metewally@ifj.edu.pl

1. Morphometric analysis of Col-HA hydrogels.

ImageJ software was applied to analyze images and evaluate the fiber diameter and pore size, manually obtained from SEM micrographs measuring fibers and pores from 10 individual images.

Supplementary Table S1. Morphometric analysis of Col-HA hydrogels (n = 200-215 fibers or pores analyzed and expressed as mean ± standard deviation).

Collagen	Concentration mg/mL	HA	Pore size µm	Fiber size nm	p-value*
PCol	3	None	0.204 ± 0.079	101.7 ± 27.4	
TCol	10	None	0.242 ± 0.122	62.9 ± 17.9	$p < 0.001$
PCol	3	Thiol-HA	2.03 ± 1.35	101.8 ± 53.4	
TCol	10	Thiol-HA	0.546 ± 0.473	76.4 ± 24.7	$p < 0.001$
PCol	3	fHA	0.201 ± 0.082	67.9 ± 23.9	
PCol	5	fHA	0.242 ± 0.074	85.6 ± 27.2	$p < 0.001$
PCol	10	fHA	0.202 ± 0.069	92.1 ± 31.7	$p < 0.001$
TCol	10	fHA	0.49 ± 0.194	85.8 ± 30.9	$p < 0.001$

*in relation to PCol (3 mg/mL), by one-way ANOVA.
TCol – telocollagen type I, native collagen form
PCol – atelocollagen type I, obtained by enzymatic digestion of TCol
fHA – fluorescein modified hyaluronic acid (HA)
Thiol-HA – thiol modified HA

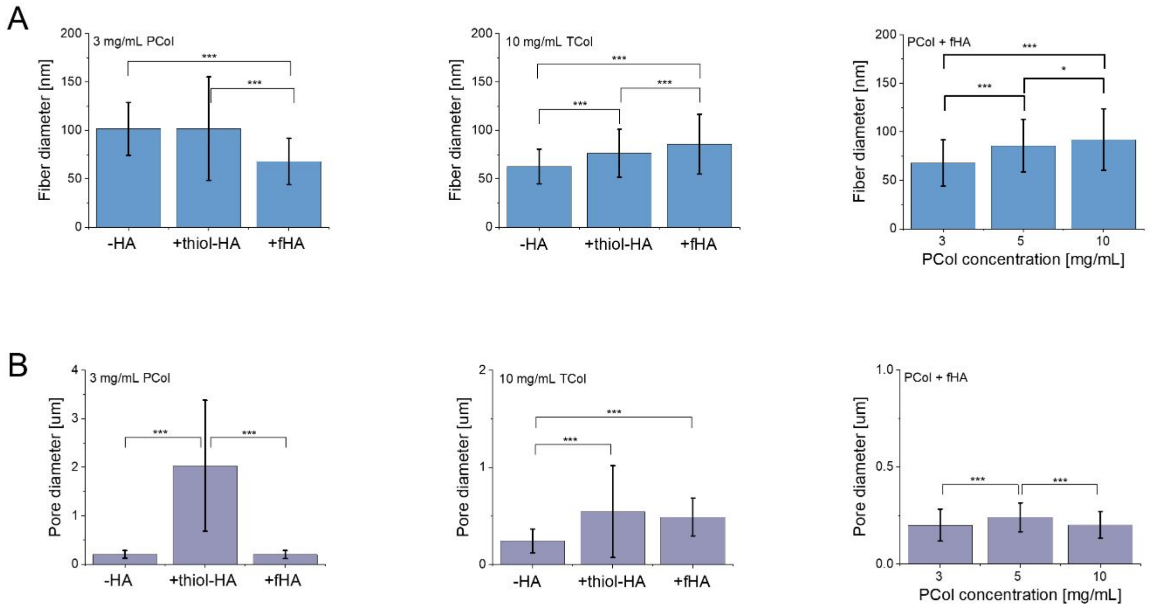

Supplementary Figure S1. Morphometric analysis of collagen hydrogels, i.e., fibril diameter (A) and pore size (B). Data are presented as a mean ± standard deviation from n = 200-215 fibers. Statistical significance was calculated using a one-way ANOVA at α = 0.05.

2. Rheological properties of Col-HA hydrogels.

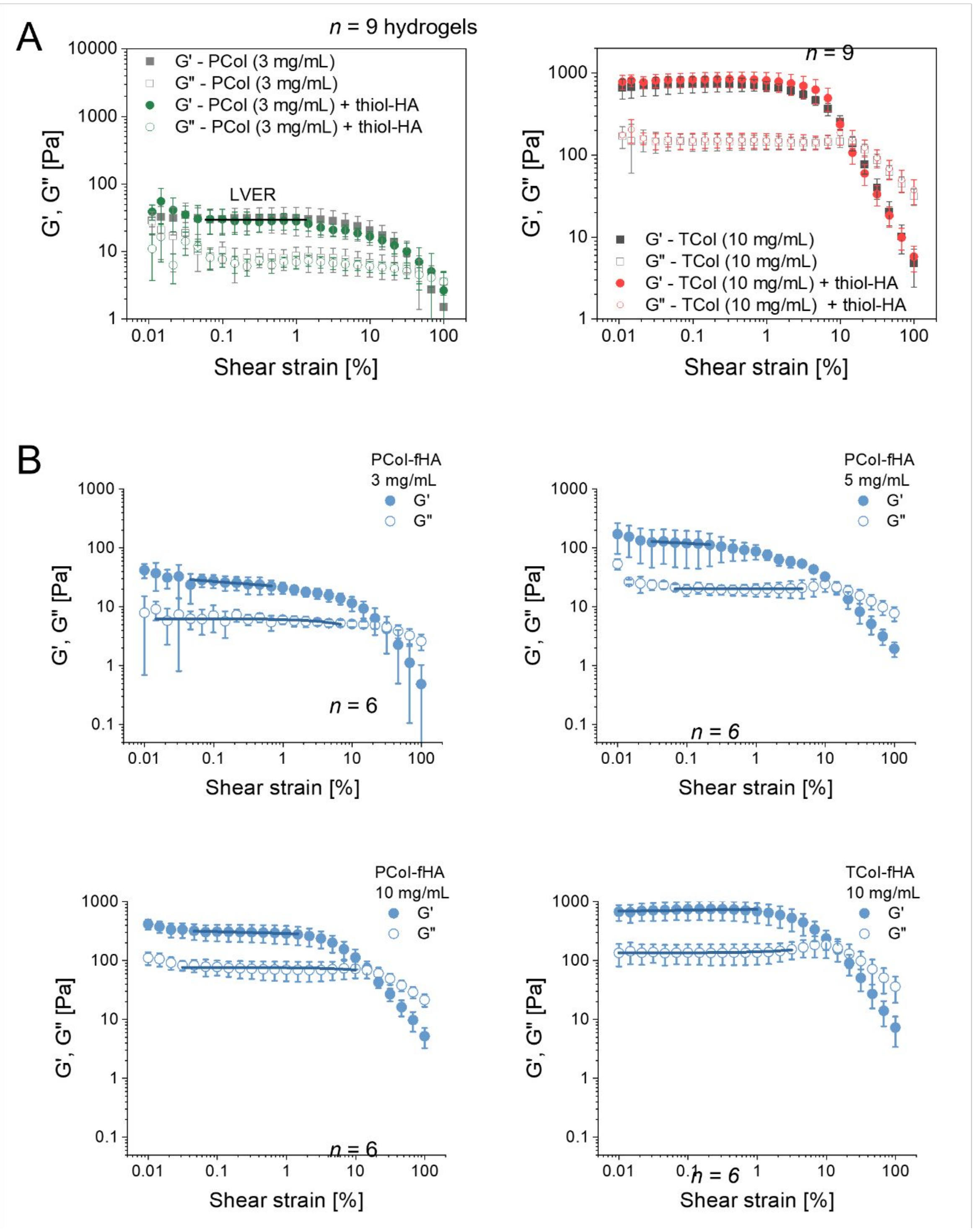

Supplementary Figure S2. Rheological properties of Col-HA hydrogels. A&B) The obtained relations between storage (G') or loss (G") moduli and shear strain were plotted within the range between 0.01 to 100%, in which lines denote the LVER region used to calculate *G'* and loss *G"*. Each point is a mean ± standard deviation of *n* individual relations recorded for each hydrogel type.

3. Viability of bladder cancer cells in spheroids

The viability of T24 (transitional cell carcinoma) cells inside spheroids embedded in various Col-HA hydrogels was obtained applying the Live/Dead Cell Double Staining Kit, which detects alive and dead cells in a population by verifying plasma membrane integrity and esterase activity. The following protocol was applied. After 3 days of spheroids incubation inside the hydrogels, culture medium was removed from samples, followed by careful rinsing with PBS. Next, 10 µl of acetoxymethyl ester of calcein (Calcein-AM) and 0.5 µl propidium iodide were mixed with 5 ml of PBS. Next, 300 µl of prepared solution was added to hydrogels containing spheroids, incubated for 15 min, and imaged immediately afterward. Calcein-AM is highly lipophilic and cell membrane permeable; therefore, it stains alive cells by emitting a green solid fluorescence (λ_{ex} = 490 nm, λ_{emi} = 515 nm). Propidium iodide is unable to penetrate the cell membrane of living cells. It stains the DNA double helix inside the nucleus by passing through the disintegrated cell membrane in dead cells by emitting red fluorescence (λ_{ex} = 535 nm, λ_{emi} = 617 nm). Fluorescence images were captured with an inverted optical microscope Olympus IX83 (Olympus, Japan) with a 10× objective (UPL FLN2 10×/0.3), U-FBW/ U-FGW filters, and the CMOS camera type Prime BSI 166 Express Scientific (01-prime-BSI-EXP, Photometrics). A 100W mercury lamp was applied to excite fluorescent dye.

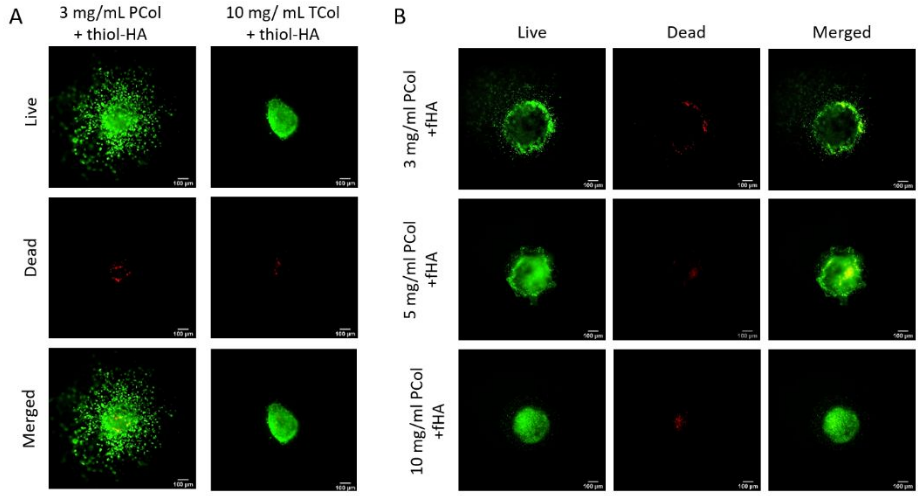

Supplementary Figure S3. Viability of bladder cancer T24 cells inside all studied Col-HA hydrogels, verified with live/dead assay. Green fluorescence (calcein-AM) shows live cells, while red fluorescence (propidium iodine) indicates dead cells.

The results show a low number of dead cells inside spheroids embedded in Col-HA hydrogels, indicating these hydrogels are suitable for growing spheroids.

4. Migration of bladder cancer cells inside 3D Col-HA hydrogels

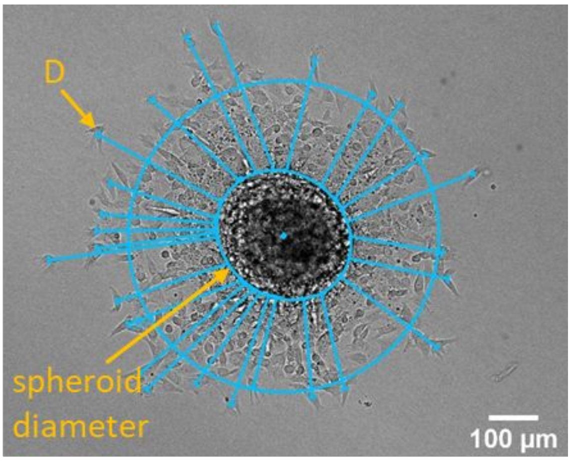

Supplementary Figure S4. Graphical representation showing the determination of a maximum distance *D* reached by a single cell migrating from the spheroid.

Supplementary Table S2. Migration of T24 cells inside Col-HA hydrogels (*n* = 124 – 144 cells analyzed and expressed as a mean ± standard deviation).

Collagen	Concentration mg/mL	HA	D @24h µm	D @48h µm	D @72h µm
PCol	3	Thiol-HA	234.26 ± 77.65	396.07 ± 102.83	500.91 ± 100.73
TCol	10	Thiol-HA	21.67 ± 17.02	32.36 ± 22.19	42.37 ± 31.66
PCol	3 mg/mL	fHA	227.37 ± 63.48	349.91 ± 65.84	476.51 ± 79.55
PCol	5 mg/mL	fHA	57.05 ± 31.38	88.84 ± 66.41	111.88 ± 84.02
PCol	10 mg/mL	fHA	18.29 ± 7.34	34.72 ± 18.17	47.67 ± 32.85

D – maximum distance reached by a single cell migrating from the spheroid, as shown in the Suppl. Fig. S4.
TCol – telocollagen type I, native collagen form
PCol – atelocollagen type I, obtained by enzymatic digestion of TCol
fHA – fluorescein-modified hyaluronic acid (HA)
Thiol-HA – thiol modified HA

5. Comparing thiol-HA and fHA

The thiol-HA used in our experiments, according to manufacturer data, falls between 0.55-0.75 µmoles/mg, resulting in a thiolation degree between 20-30%, obtained using the Ellman's test (https://advancedbiomatrix.com/glycosil.html). The fluorescently labeled HA is characterized by a 4-8 mole% substitution degree, much lower than the HA thiolation.

6. Spearman correlation coefficient

In our data, we did not expect linear correlation due to the non-linear character of collagen-based hydrogels. Therefore, we applied Spearman correlation analysis to identify correlations among the evaluated parameters. The Spearman correlation coefficients are measures of monotonic relationships, quantifying the strength and direction of the monotonic relationship between two variables, meaning how well one variable can predict the other, even if the relationship is not linear. It does this by calculating the correlation between the ranked values of the two variables. The coefficient ranges from -1 to +1, with +1 indicating a perfect positive monotonic relationship, 0 indicating no relationship, and -1 indicating a perfect negative one.

	A(X)	B(Y)	C(Y)	D(Y)	E(Y)	F(Y)	G(Y)	H(Y)	I(Y)	J(Y)
Long Name		fiber diameter	pore size	storage modulus	loss modulus	loss tangent	migration distance @72h	HA type	Col concentration	Col type
Units										
Comments	Spearman Correlations	Spearman Correlations	Spearman Correlations	Spearman Correlations	Spearman Correlations	Spearman Correlations	Spearman Correlations	Spearman Correlations	Spearman Correlations	Spearman Correlations
F(x)=										
1	fiber diameter	1	0.6	0.1	0.1	-0.1	0.3	-0.28868	0	-0.35355
2	pore size	0.6	1	0.3	0.3	-0.3	0.1	-0.86603	0	0.35355
3	storage modulus	0.1	0.3	1	1	-0.7	-0.9	-0.28868	0.94868	0.70711
4	loss modulus	0.1	0.3	1	1	-0.7	-0.9	-0.28868	0.94868	0.70711
5	loss tangent	-0.1	-0.3	-0.7	-0.7	1	0.6	0	-0.63246	-0.35355
6	migration distance @72h	0.3	0.1	-0.9	-0.9	0.6	1	0	-0.94868	-0.70711
7	HA type	-0.28868	-0.86603	-0.28868	-0.28868	0	0	1	0	-0.61237
8	Col concentration	0	0	0.94868	0.94868	-0.63246	-0.94868	0	1	0.55902
9	Col type	-0.35355	0.35355	0.70711	0.70711	-0.35355	-0.70711	-0.61237	0.55902	1

Supplementary Figure S4. The output of the Spearman correlation analysis (obtained using OriginPro 2022 software). Significant correlations are marked in red. A positive correlation between storage/loss modulus and collagen concentration indicates that increased collagen content leads to stiffening of the hydrogels, and simultaneously, the resulting stiffness inhibits the bladder cancer cell migration (negative correlation).
